# Evaluating endometrial response to human chorionic gonadotropin: alterations in epigenetic regulation and extracellular vesicle cargo of endometrial stromal cells

**DOI:** 10.1093/hropen/hoaf051

**Published:** 2025-08-14

**Authors:** Deimantė Žukauskaitė, Erika Girniūtė, Rūta Navakauskienė

**Affiliations:** Department of Molecular Cell Biology, Institute of Biochemistry, Life Sciences Center, Vilnius University, Vilnius, Lithuania; Department of Molecular Cell Biology, Institute of Biochemistry, Life Sciences Center, Vilnius University, Vilnius, Lithuania; Department of Molecular Cell Biology, Institute of Biochemistry, Life Sciences Center, Vilnius University, Vilnius, Lithuania

**Keywords:** endometrium, epigenetics, histone modification, miRNA, extracellular vesicles, hCG

## Abstract

**STUDY QUESTION:**

What is the effect of hCG on the epigenetic profile and the expression of other molecular factors in endometrial stromal cells (ESCs)?

**SUMMARY ANSWER:**

Our findings suggest that hCG treatment alters the molecular environment of decidualized ESCs, potentially influencing implantation and immune regulation through epigenetic modifications and changes in the levels of secreted proteins and micro-ribonucleic acids (miRNAs).

**WHAT IS KNOWN ALREADY:**

Embryo implantation depends not only on the quality of the embryo but also on the receptivity of the endometrium, the specialized lining of the uterus that undergoes dynamic changes to support pregnancy. Effective communication between the maternal and fetal compartments, facilitated by molecular signals and cellular interactions, is essential for successful implantation.

**STUDY DESIGN, SIZE, DURATION:**

Cross-sectional study of patient-derived ESCs comparing untreated cells with cells treated with hCG and/or decidualization induction. The number of samples depends on the method and varies from 2 to 8. Results were analyzed after 6-, 24-, 48-, and 72-h time-points.

**PARTICIPANTS/MATERIALS, SETTING, METHODS:**

ESCs were isolated from patients undergoing assisted reproductive technologies. In the study, we analyzed changes in the epigenetic profile and other molecular factors of ESCs during decidualization and in *in vitro* response to the embryo-secreted factor, hCG. ESCs were induced for decidualization for 3 days (medroxyprogesterone acetate+cAMP), or treated with hCG for 24 h, or given combined treatment: 2 days of decidualization followed by 24 h of hCG. Furthermore, we compared decidualized ESCs with decidualized ESCs that were also treated with hCG. We examined various cellular properties, including morphology, metabolic activity, and cell viability of ESCs after induction of decidualization and hCG treatment. Additionally, we assessed changes in the expression of genes associated with decidualization, inflammatory response, apoptosis regulation, and epigenetic factors using RT-qPCR. The levels of histone modifications and the factors regulating these modifications were explored by performing western blot assays. Additionally, we performed a chromatin immunoprecipitation assay to extract gene regions enriched with the epigenetic modification H3K27Ac. Finally, we analyzed the protein and miRNA level changes in ESC extracellular vesicles (ESC-EVs) after the indicated treatments, using mass spectrometry and small RNA sequencing.

**MAIN RESULTS AND THE ROLE OF CHANCE:**

Our study found that hCG treatment increased prolactin gene (*PRL)* expression (*P* < 0.05), while the expression of *IL6* and *BAK1* was inhibited in ESCs (*P* < 0.05). We also revealed that hCG affects epigenetic regulation, leading to changes in the expression of *EED*, *HDAC1*, and *TET1/2/3* (*P* < 0.05). Specifically, hCG treatment resulted in increased levels of H3K27Ac in gene regions associated with decidualization, such as *FOXO1* (*P* < 0.05), and implantation genes like *HOXA10* and *HAND2* (*P* = 0.06). After decidualization, we observed increased protein levels in ESC-EVs that are associated with embryo implantation (*P* = 0.0038) and pregnancy (*P* = 0.0012). These included proteins such as FIBL-1, IGFBP-1/7, MMP-2, STC-1/2, and PAPP-A (*P* < 0.05). Additionally, hCG treatment in decidualized ESCs elevated the levels of proteins involved in immune system regulation, including PR-3 (*P* = 0.0131). Moreover, we revealed changes in miRNA levels within EVs secreted by ESCs following hCG treatment and decidualization, which are associated with embryo development in the uterus (*P* = 0.03). Notable miRNAs include hsa-miR-340-3p, hsa-miR-663a, hsa-miR-766-5p, hsa-miR-3138, and hsa-miR-3180-5p (*P* < 0.05).

**LARGE SCALE DATA:**

N/A.

**LIMITATIONS, REASONS FOR CAUTION:**

This study utilizes a comprehensive analysis to explore potential epigenetic and molecular targets essential for endometrial function. The research is based on *in vitro* experiments using ESCs derived from *ex vivo* samples. Future studies involving a broader range of cell types and larger sample sizes could help to further validate and expand upon these results.

**WIDER IMPLICATIONS OF THE FINDINGS:**

We found that hCG enhances the decidualization process in a dose-dependent manner and affects implantation and immune regulation through epigenetic changes, as well as variations in the levels of secreted proteins and miRNAs. Our study suggests that the application of hCG in assisted reproduction technologies may offer potential benefits for patients. However, carefully considering the appropriate dosage is important to ensure optimal outcomes.

**STUDY FUNDING/COMPETING INTEREST(S):**

This research did not receive any specific grant from funding agencies in the public, commercial, or not-for-profit sectors. The authors have no conflicts of interest to declare.

WHAT DOES THIS MEAN FOR PATIENTS? The inner lining of the uterus, known as the endometrium, plays a crucial role in successful implantation. This lining experiences monthly changes throughout the menstrual cycle and undergoes endometrial cell transformation into a more specific cell type required for implantation and pregnancy, which is influenced by sex hormones produced by the ovaries. During the preimplantation period, it is essential for the endometrium to appropriately respond to signals from the embryo and ensure a receptive state preparation. Molecular changes in endometrial cells may contribute to cases of unexplained infertility. Epigenetics is the study of how cells regulate gene activity without changing the DNA sequence. Epigenetic mechanisms govern the changes in cells that affect their functioning. We revealed that the hormone human chorionic gonadotropin (hCG) induces specific epigenetic modifications associated with an increase in various gene activities. Therefore, modifying these epigenetic markers could enhance the receptivity of the endometrium. Additionally, most cells, including endometrial cells and embryos, communicate by secreting and exchanging vesicles—small particles released by cells that help to communicate with each other, containing various biological molecules. We identified specific biological molecules that endometrial cells release in response to hCG (the embryo’s signal), and these molecules are integral to the processes of embryo implantation, including immunoregulation and pregnancy development. The therapeutic application of these biomolecules, encapsulated in vesicles, could also be beneficial in improving fertility, and these molecules could also serve as diagnostic markers.

## Introduction

The endometrium is the inner lining of the uterus, essential for the successful attachment of the embryo and its subsequent development during pregnancy. A critical aspect of preparing the endometrium for receptivity during each menstrual cycle is the differentiation of endometrial stromal cells (ESCs) into decidual cells, which is induced by progesterone (Okada *et al.*, 2018; Ng *et al.*, 2020). When conception occurs, the embryo begins to secrete factors that influence the endometrium’s readiness for implantation. One of the earliest signals indicating the presence of an embryo is hCG, which is secreted by trophoblast cells (d’Hauterive *et al.*, 2022). Studies have shown that hCG affects the endometrium through luteinizing hormone/choriogonadotropin receptors (LHCGRs) by increasing intracellular cAMP levels (Tapia-Pizarro *et al.*, 2017; Sacchi *et al.*, 2018). It also regulates various processes, including tissue vascularization (Xu *et al.*, 2020), extracellular matrix modulation (Fluhr *et al.*, 2008; Tapia-Pizarro *et al.*, 2013; Riboldi *et al.*, 2020), immunoregulation (Bourdiec *et al.*, 2013), decidualization (Koch *et al.*, 2018), and implantation (Makrigiannakis *et al.*, 2017; Zhu *et al.*, 2020). Furthermore, research has indicated that intrauterine infusion of hCG helps synchronize the glandular and stromal compartments of the endometrium (Strug *et al.*, 2016). Despite these findings, a recent study has demonstrated that only a small subpopulation of ESCs expresses LHCGRs. It was also suggested that hCG might not be able to elevate cAMP levels or induce the expression of decidualization markers (Mann *et al.*, 2022). Additionally, another study found that prolonged exposure to low doses of hCG in endometrial epithelial cells resulted in the downregulation of LHCGRs (Evans & Salamonsen, 2013).

Conflicting results have been observed in the clinical analysis of the effects of intrauterine hCG application on pregnancy rates during assisted reproductive technologies. Some studies have reported a positive influence of hCG (Bielfeld *et al.*, 2019; Liu *et al.*, 2019; Wadhwa & Rani, 2021; Jahanshahi *et al.*, 2022), while others found no beneficial effects (Shiotani *et al.*, 2017; Hou *et al.*, 2018; Abdallah *et al.*, 2021). These discrepancies may be due to variations in the protocols and concentrations of hCG used in different studies. The conflicting outcomes highlight the need for a more thorough evaluation of hCG’s impact on endometrial cells’ properties.

Epigenetics refers to heritable mechanisms that regulate gene expression without altering the DNA sequence. It is well-established that epigenetic mechanisms, including DNA methylation, histone modifications, and micro-ribonucleic acids (miRNAs), play significant roles in fundamental cellular processes (Dupont *et al.*, 2009; Deans and Maggert, 2015). Previous research has shown that hCG modulates the immunological mechanisms in the endometrium by increasing the levels of the repressive chromatin mark H3K27Me3 and its regulator, EZH2 (Silasi *et al.*, 2020). Furthermore, hCG has been found to downregulate *HOXA10* promoter methylation (Zhu *et al.*, 2020). These findings emphasize the importance of epigenetic modifications in the regulatory effects of hCG on the endometrium.

Alterations in the communication between the maternal environment and the embryo before implantation may contribute to pregnancy complications, such as miscarriage and recurrent implantation failure (Idelevich & Vilella, 2020; Günther *et al.*, 2023). Recent studies have demonstrated that embryo-derived extracellular vesicles (EVs) can convey information about embryo quality to oviductal epithelial cells (Dissanayake *et al.*, 2021). Additionally, both embryos and the endometrium have the potential to secrete EVs, which facilitates paracrine signaling and prepares other endometrial cells for implantation (Mishra *et al.*, 2021). Therefore, a comprehensive analysis of epigenetic regulation in embryo signaling (via hCG) and endometrial preparation (through decidualization) could help identify potential targets associated with implantation difficulties. We investigated the impact of decidualization and hCG influences on the cellular, molecular, and epigenetic properties of ESCs, including their metabolic activity, viability, decidualization response, and histone modifications, as well as the protein and miRNA profiles of EVs secreted by these cells. Our findings indicate that hCG alters the molecular environment of decidualized ESCs, which may play a significant role in embryo implantation and immune regulation through epigenetic modifications and changes in the levels of secreted proteins and miRNAs.

## Materials and methods

### Ethical approval

This study has received approval from the Vilnius Regional Biomedical Research Ethics Committee under the reference number 158200–18/7–1049–550.

### Tissue samples collection and ESC isolation

In preparation for assisted reproductive technology, endometrial biopsy procedures were performed by employing endometrial scratching with a sterile plastic tube on women undergoing IVF at Vilnius University Hospital Santaros Klinikos, Obstetrics and Gynaecology Center, Santaros Fertility Center. The samples of endometrial tissue were collected during the luteal phase (Days 17–22) of the menstrual cycle. All participants in this research provided informed consent for their involvement in the study.

Collected endometrial biopsies were washed a few times with sterile phosphate-buffered saline (PBS) (Gibco, Thermo Fisher Scientific, Waltham, MA, USA). Subsequently, ESCs were isolated from the biopsies following the established method (Tavakol *et al.*, 2018). The growth medium for cultivation consisted of DMEM/F12 (Gibco, Thermo Fisher Scientific) supplemented with 10% fetal bovine serum (FBS) (Gibco, Thermo Fisher Scientific), 100 U/ml penicillin, and 100 µg/ml streptomycin (Genaxxon Bioscience GmbH, Ulm, Germany). The cells were cultivated at 37 °C with 5% CO_2,_ with passaging every 2–3 days. Early passages (3–5 passages) of cell culture were used for all following experiments.

### Induction of decidualization and treatment with hCG

To evaluate decidualization inducers and hCG impact, cells were seeded into cultivation flasks using a growth medium and left to grow until they reached around 80% confluence. The growth medium was aspirated, and decidualization medium (RPMI) (without phenol) (Hyclone Laboratories, Logan, UT, USA); 2% charcoal-treated FBS (Thermo Fisher Scientific); 100 U/ml penicillin; 100 µg/ml streptomycin; 0.5 mM dibutyryl-cAMP (db-cAMP) (Selleckchem, Houston, TX, USA); 1 µM medroxyprogesterone acetate (dissolved in DMSO, whose final concentration in solution was 0.1%) (Cayman Chemical Company, Ann Arbor, MI, USA) or induction medium (decidualization medium without decidualization inductors) was incubated on the cells for 2 days. After 2 days, 10 or 50 IU/ml (for 3-(4,5-dimethylthiazol-2-yl)-2,5-diphenyltetrazolium bromide (MTT) assay and viability assessment, 100 IU/ml was also included) concentration of human urine-derived hCG (Sigma-Aldrich, St Louis, MO, USA) was added to appropriate flasks. Unless stated otherwise, all reagents were reconstituted in water. At the same time, samples without hCG treatment received no additions, and after an additional 6 or 24 h, all cells were collected for the following analysis.

### MTT assay and viability assessment

For metabolic efficiency evaluation, cells were seeded in 48-well plates at a density of 3 × 10^4^ cells per well and cultured with various induction media (as described in the sub-section ‘Induction of decidualization and treatment with hCG’) for 24, 48, and 72 h. Following the respective incubation periods, the cell culture medium was aspirated, and to assess cell viability, 0.2 mg/ml MTT reagent (Sigma-Aldrich) was added to each well, followed by a 1-h incubation at 37 °C. The resulting formazan precipitate was dissolved in 96% ethanol, and the optical density of each well was measured using an Infinite^®^ M200 Pro plate reader spectrophotometer (Tecan, Mannedorf, Switzerland) at 570 and 630 nm wavelengths. The absorbance at 570 nm represents the formazan product, while the 630-nm wavelength serves as a reference. The absorbance results of the MTT assay are presented as the difference between values at 570 and 630 nm.

For viability assessment, the conditioned medium was centrifuged to collect detached cells, and these cells were then combined with those collected using trypsin (Thermo Fisher Scientific). Subsequently, the cells were stained with Trypan Blue (Thermo Fisher Scientific), which targets dead cells, and both stained and non-stained cells were counted using a hemacytometer. Viability (%) was calculated as the percentage of viable (non-stained) cells relative to the total number of cells (stained and non-stained).

### Total RNA extraction and RT-qPCR

Total RNA was extracted from ESCs using the Quick-DNA/RNA™ Miniprep Kit (Zymo Research, Irvine, CA, USA) following the manufacturer’s instructions. The purity and concentration of RNA were assessed using the Implen NanoPhotometer™ (Implen GmbH, Munich, Germany). Subsequently, up to 1 μg of RNA was taken for RT to generate complementary DNA using LunaScript^®^ RT SuperMix Kit (New England Biolabs, Ipswich, MA, USA). Quantitative polymerase chain reaction (qPCR) was performed using the Luna^®^ Universal qPCR Master Mix (New England Biolabs, Ipswich, MA, USA) on the RotorGene 6000 thermocycler with Rotor-Gene 6000 series software (Corbett Life Science, QIAGEN, Hilden, Germany). The geometric mean of threshold cycle (C_T_) values for *GAPDH* and *RPL13A* was employed to normalize mRNA levels. Detailed primer sequences can be found in Supplementary Table S1.

### ELISA

The cell culture media following treatments described in ‘Induction of decidualization and treatment with hCG’ were collected to evaluate secreted prolactin. The Human Prolactin ELISA kit (Biovendor—Laboratorni medicina a.s., Karasek, Czech Republic) was employed following the manufacturer’s instructions. The total amount of proteins in the analyzed samples was quantified using a Bradford Assay Kit (Thermo Fisher Scientific).

### Western blot analysis

Immunoblot analysis was conducted following the methodology described in prior studies (Žukauskaitė *et al*., 2024). Primary antibodies used in this analysis were diluted as per the manufacturer’s recommendations: GAPDH (Abcam (Cambridge, UK) Cat No. ab8245, RRID: AB_2107448), EZH2 (Thermo Fisher Scientific Cat No. PA5-29129, RRID: AB_2546605), H3K4me3 (Millipore Cat No. 05-745R, RRID: AB_1587134), H3K9me3 (Millipore (Burlington, MA, USA) Cat No. 07-442, RRID: AB_310620), H4hyperAc (Millipore Cat No. 06-946, RRID: AB_310310), H3K9Ac (Cell Signaling Technology (Danvers, MA, USA) Cat No. 9649, RRID: AB_823528), HDAC1 (Santa Cruz Biotechnology (Dallas, TX, USA) Cat No. sc-81598, RRID: AB_2118083), and HDAC2 (Santa Cruz Biotechnology Cat No. sc-9959, RRID: AB_627704), H3K27ac (Cell Signaling Technology Cat No. 8173 (also ENCAB502OHI), RRID: AB_10949503), H3K27me3 (Millipore Cat No. 07-449, RRID: AB_310624), SUZ12 (Cell Signaling Technology Cat No. 3737, RRID: AB_2196850), EED (Thermo Fisher Scientific Cat No. MA5-16314, RRID: AB_2537833). Horseradish peroxidase-conjugated secondary antibodies targeting mouse and rabbit immunoglobulins (Advansta, San Jose, CA, USA) were employed. Chemiluminescent detection utilized the WesternBright™ Quantum detection kit (Advansta), and signal capture was performed using the ChemiDoc™ XRS+ System (BIO-RAD, Hercules, CA, USA). β-actin, histone H3, and H4 were employed as loading controls, and quantitative analysis of the detected protein band density was carried out using ImageJ software (National Institutes of Health, Bethesda, MD, USA).

### ChIP-qPCR

Chromatin immunoprecipitation (ChIP) assays were conducted with a Zymo-Spin ChIP Kit (Zymo Research) according to the manufacturer’s protocol with some modifications as described earlier (Žukauskaitė *et al*., 2023). In this study for ChIP assays, we used ChIP-grade antibodies against H3K27Ac (Cell Signaling Technology Cat No. 8173 (also ENCAB502OHI), RRID: AB_10949503) and H3K27Me3 (Millipore Cat No. 17-622, RRID: AB_916347) and a nonspecific IgG-control antibody (Advansta). Details of qPCR primers used for targeting specific promoter and exon regions are provided in Supplementary Table S2.

The enrichment of a specific histone modification on a particular region was calculated as a percentage of Input values using the following formulae:

$$\Delta Ct=((Ct\;[\mathrm{Input}\;\mathrm{DNA}]-\;{\mathrm{Log}}_{2}(\mathrm{Input}\;\mathrm{Dilution}\;\mathrm{Factor}))-Ct\;[\mathrm{ChIP}\;\mathrm{DNA}])$$

 

$$\%\;of\;\mathrm{Input}\;=100\%\;\times \;2^{\Delta Ct}$$

### Collection, characterization, and total RNA isolation of small EVs

For collection of EVs, cells were treated with appropriate media as described in the sub-section ‘Induction of decidualization and treatment with hCG’ but excluding FBS from all medium composition. To collect EVs, two to three repeated preparations from the same patient samples were combined to increase the EVs’ yield. Cells were treated without FBS only for an EVs collection time of up to 3 days. EVs were collected through differential centrifugation of conditioned media, involving the following steps: 300 *g* for 10 min (to remove cells), 2000 *g* for 10 min (to collect cell debris), 20 000 *g* for 30 min (to collect larger EVs), and 100 000 *g* for 70 min (to collect smaller EVs). A washing step for the collected small EVs with PBS, followed by additional centrifugation at 100 000 *g* for 70 min, was included. All centrifugation steps were conducted using an Optima XE Ultracentrifuge (Beckman Coulter, Brea, CA, USA) with a fixed-angle rotor Type 70 TI at 4 °C. After the wash step, collected EVs were suspended in PBS and transferred to −80°C until needed. For EVs characterization immunoblot analysis was performed as described in ‘Immunoblot analysis’ using these antibodies: TSG101 (Abcam Cat No. ab125011, RRID: AB_10974262), Alix (Abcam Cat No. ab88388, RRID: AB_2042596), HSP70 (R & D Systems (Minneapolis, MN, USA) Cat No. AF1663, RRID: AB_2233171), Annexin V (R & D Systems Cat No. AF399, RRID: AB_2258445), CD9 (R & D Systems Cat No. MAB25292, RRID: AB_2910230), Cytochrome c (Novus (St Louis, MN, USA) Cat No. NB100-56503, RRID: AB_838084), GM130 (Novus Cat No. NBP2-53420, RRID: AB_2916095), CD81 (Novus Cat No. NB100-65805, RRID: AB_962702). The sizes and counts of isolated EVs were evaluated using a nanoparticle tracking analyzer, NanoSight LM10-HS (NanoSight, Salisbury, UK). Samples for scanning electron microscopy were prepared as described previously (Skliutė *et al.*, 2024). Total RNA was isolated from EVs suspended in PBS using TRIzol™ reagent (Invitrogen, Thermo Fisher Scientific) according to the manufacturer’s instructions.

### Protein identification by mass spectrometry

The identification of proteins in EVs using mass spectrometry was performed by the Proteomics Department at the University of Bristol (Bristol, UK). Peptides from different samples were first labeled with distinct TMT (Tandem Mass Tag) molecules and then combined for further analysis. High pH reversed-phase chromatography was used to fractionate the peptides, followed by Liquid Chromatography Tandem Mass Spectrometry analysis. The obtained data were aligned with the human UniProt database (https://www.uniprot.org/), and protein grouping was determined using PD2.4 software (Thermo Fisher Scientific). Protein quantities in each sample were normalized to the total protein content of the sample and log-transformed (log2) to approximate a normal distribution. Two biological replicates (n = 2) were performed, with protein levels determined separately for each and fold changes calculated between conditions. The final results represent the average fold changes from both replicates. For proteomic analysis FactoMineR package (Le S *et al.*, 2008) was applied. Normalized values were used for statistical analysis, and group comparisons were performed using a paired *t*-test. *P*-values were adjusted using the Benjamini–Hochberg method. A protein interaction network was generated using the STRING functional association analysis tool (https://string-db.org/), including proteins with log2 (fold change) >1.2 and *P* < 0.1. The STRING tool was also used to assign altered proteins to biological processes (GO) and functional pathways (KEGG).

### Small RNA sequencing

Small RNA sequencing and bioinformatic analysis services were provided by Novogene Co., Ltd (Beijing, China). Three biological replicates (n = 3) were performed. Library preparation and sequencing were performed using the Illumina NovaSeq 6000 platform in SE50 mode, generating 18–40 bp reads. Bowtie (Langmead *et al.*, 2009) was employed to map small RNA tags to the reference sequence. The prediction of miRNA target genes was conducted using miRanda. miRNA expression levels were evaluated using transcript per million with the normalization formula: Normalized expression = mapped read count/Total reads × 1 000 000, following the criteria outlined by Zhou *et al.* (2010). Differential expression analysis between two samples utilized the DEGseq2 R package (Love *et al.*, 2014) with *P*-values adjusted using the Benjamini & Hochberg method. A corrected *P*-value of 0.05 was set as the threshold for significantly differential expression by default. Gene ontology (GO) enrichment analysis was performed on the target gene candidates of differentially expressed miRNAs. KOBAS (Mao *et al.*, 2005) software was also employed to assess the statistical enrichment of target gene candidates in KEGG pathways.

### Statistical analysis

GraphPad Prism 8 (GraphPad Software Inc., San Diego, CA, USA) was employed for statistical analysis. The results are presented as medians with interquartile range, unless specified otherwise. To evaluate significant differences between the tested groups, for unpaired samples, a nonparametric Mann–Whitney test, and for paired samples Wilcoxon test was applied, with a significance level (α value) set at 0.05. Statistical significance is denoted as * for *P*-values <0.05 and ** for *P*-values <0.01.

## Results

### hCG impact on ESC properties and decidualization process

**Figure 1.**
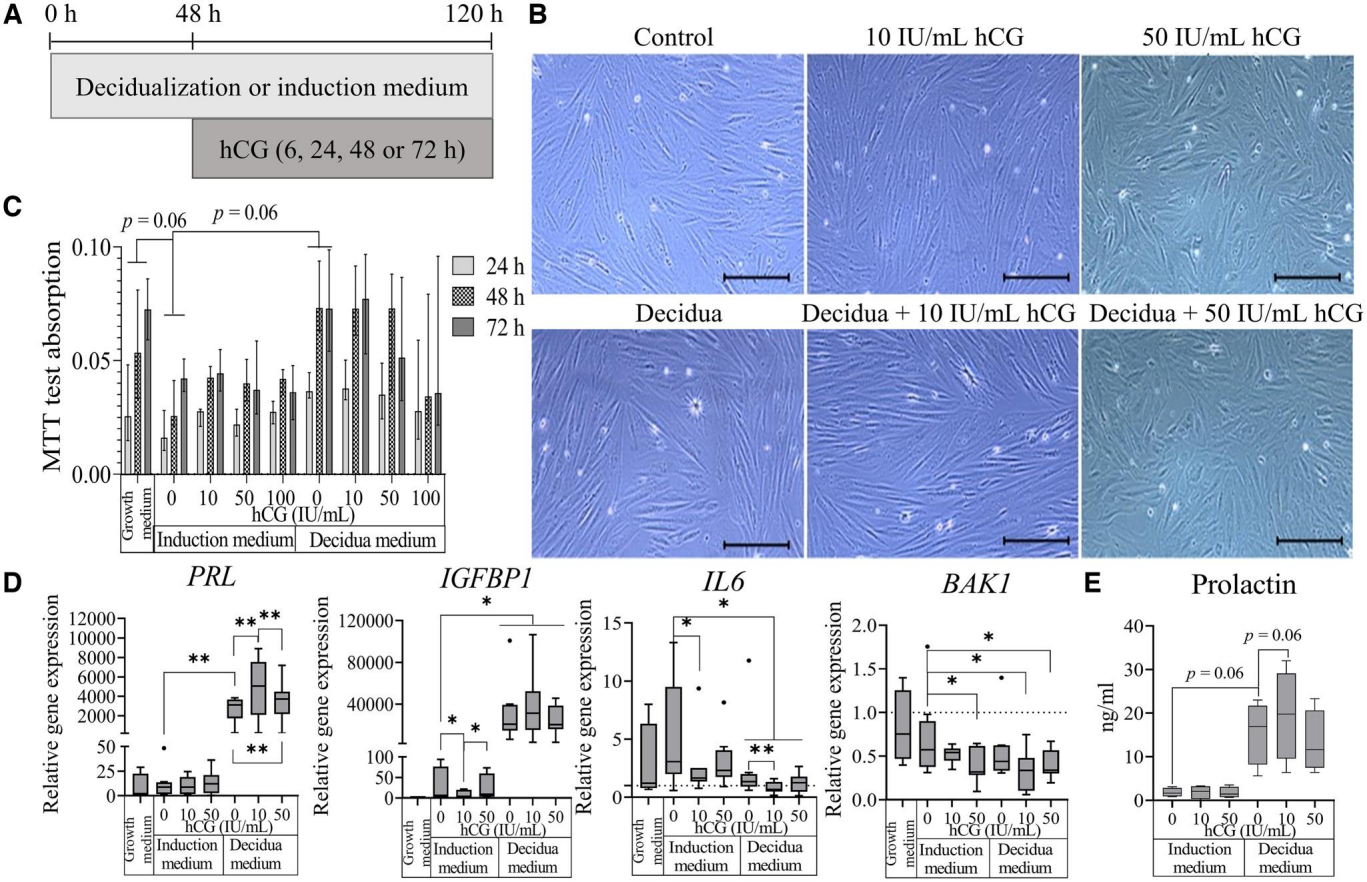
**Assessment of the impact of hCG on endometrial stromal cell (ESC) properties and decidualization process.** (**A**) Schematic representation of experiment design. (**B**) Evaluation of ESC morphology after decidualization (decidua) and hCG treatments. The scale bar represents 400 µm. (**C**) Metabolic activity assessed using the MTT test. Results are presented as sample absorbance values minus blank values (570–630 nm) and are shown as the median with interquartile range (n = 4). (**D**) Gene expression changes after decidualization and hCG treatments confirmed by RT-qPCR. Results were calculated using the ΔΔC_T_ method (n = 8). The geometric mean of *GAPDH* and *RPL13A* expression levels was used for mRNA level normalization. (**E**) Quantification of prolactin levels secreted by ESCs, detected by ELISA. Protein levels were normalized to total protein quantity, measured using the Bradford assay (n = 4). Results are presented as box plots, where the middle line represents the median value, the box represents the interquartile range, and error bars extend to the most extreme data points that are not considered outliers. *P-value <0.05; **P-value <0.01. *P*-values were calculated using the Wilcoxon test.

Our experimental setup aimed to evaluate the effect of hCG on ESCs previously cultured for 48 h in either decidualization medium or induction medium without decidualization, followed by the addition of hCG for 6, 24, 48, and 72 h (Fig. 1A). No changes in fibroblast-type cell morphology were observed following the analyzed treatments (Fig. 1B). An MTT test revealed that ESCs treated with only an induction medium exhibited decreased metabolic activity, compared with ESCs cultivated in a standard growth medium (Fig. 1C). However, when the induction medium was supplemented with decidualization inducers (decidualization medium), the observed inhibition of ESC metabolic activity was diminished. Furthermore, no significant impact of different hCG concentrations on metabolic activity was detected, except for the visualized tendency of lower results in previously decidualized ESCs treated with 100 IU/ml hCG after 48 h, compared to only decidualized cells. Meanwhile, assessment of ESC viability by staining cells with Trypan Blue revealed no negative effect on viability, neither of different hCG concentrations alone, nor in combination with previously decidualized cells (Supplementary Fig. S1A). So, the results of these findings confirmed that selected hCG concentrations had no negative impact on ESC morphology, metabolic activity, and viability. For the following analysis, 10 and 50 IU/ml 24-h hCG treatment was selected.

Next, we evaluated the effect of hCG on known markers of the decidualization process, such as prolactin (PRL) and insulin-like growth factor-binding protein 1 (IGFBP-1). Analysis of these markers’ gene expression confirmed that decidualization induction led to significant upregulation of PRL and IGFBP-1 mRNA levels compared to the induction medium (Fig. 1D). Although adding hCG to non-decidualized ESCs did not affect *PRL* gene expression, we demonstrated that 10 IU/ml hCG increased *PRL* expression in previously decidualized ESCs. However, a higher concentration of hCG (50 IU/ml) led to a significantly lower increase in *PRL* expression compared to 10 IU/ml, although the *PRL* expression levels remained significantly higher than in the group of only the decidualized ESCs. Meanwhile, 10 IU/ml hCG was associated with a downregulation of *IGFBP1* expression in non-decidualized cells. However, this concentration also demonstrated a positive regulatory tendency in decidualized cells, but the change was not statistically significant. Our results confirmed that decidualization induction in ESCs was associated with the downregulation of *IL6*, and this decrease was significantly reduced by adding 10 IU/ml hCG in both non-decidualized and decidualized ESCs. Subsequently, hCG treatment did not affect the expression of *IL1B* and *IL1*, while the expression of both these interleukin-coding genes increased after decidualization induction (Supplementary Fig. S1B). Despite not observing changes in ESC viability, we also analyzed apoptosis-associated genes’ expression altered by hCG and decidualization stimulus. The results revealed that decidualization stimulus alone was able to inhibit *BAX* and *BCL* expression (Supplementary Fig. S1C). Furthermore, we demonstrated the impact of hCG treatment on the pro-apoptotic process, as 10 IU/ml hCG in decidualized ESCs and 50 IU/ml hCG in both non-decidualized and decidualized ESCs led to downregulation of *BAK1* expression (Fig. 1D). Moreover, the observed positive impact of 10 IU/ml hCG on prolactin expression in decidualized ESCs was confirmed at the protein level (*P* = 0.06) by evaluating the amount of prolactin secreted into the conditioned medium using ELISA (Fig. 1E). These results revealed that hCG treatment has a dose-dependent impact on the decidualization process, with 10 IU/ml leading to the highest enhancement and influencing inflammation and apoptosis regulation.

### hCG impact on ESC epigenetic regulation

**Figure 2.**
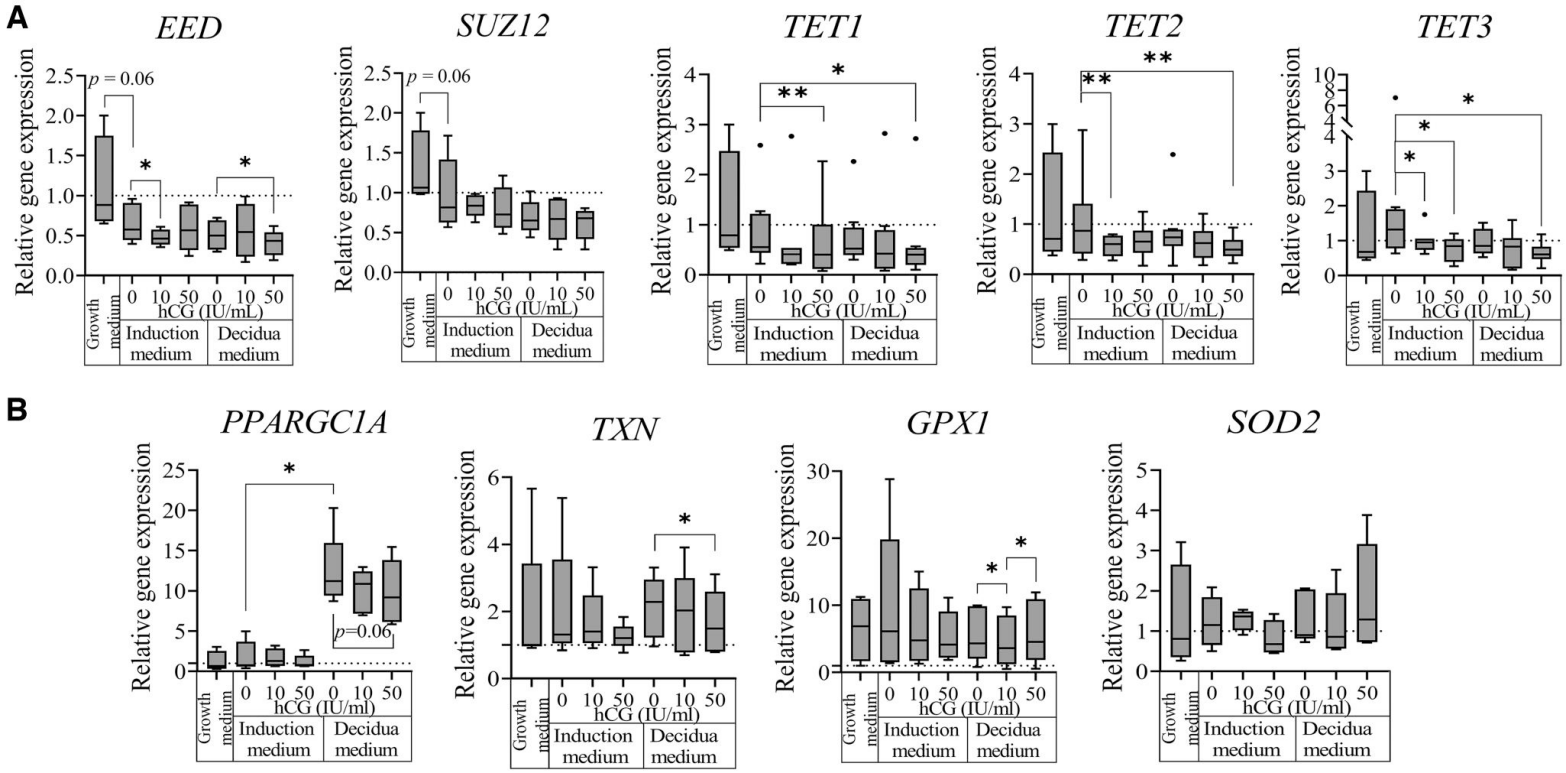
**Gene expression changes induced by decidualization and hCG treatment of ESCs.** (**A**) Epigenetic modulators of histone modifications, and DNA demethylation factors. (**B**) Metabolism-associated factors. Gene expression changes were analyzed using RT-qPCR. Results were calculated using the ΔΔC_T_ method (n = 8). mRNA levels were normalized to the geometric mean of *GAPDH* and *RPL13A* expression. Results are presented as box plots, where the middle line represents the median value, the box represents the interquartile range, and error bars extend to the most extreme data points that are not considered outliers. *P-value <0.05; **P-value <0.01. *P*-values were calculated using the Wilcoxon test. ESCs, endometrial stromal cells.

Furthermore, we analyzed changes in the gene expression of various epigenetic regulators induced by hCG in ESCs. A 10-IU/ml hCG concentration in non-decidualized ESCs and 50 IU/ml in previously decidualized cells led to the inhibition of *EED* expression, while *SUZ12* was not significantly affected (Fig. 2A). hCG also affected DNA demethylases' gene expression, as our results showed that a 50-IU/ml hCG concentration inhibited *TET1*, *TET2*, and *TET3* expression in both non-decidualized and decidualized ESCs. In addition, 10 IU/ml hCG in non-decidualized cells was able to significantly downregulate *TET2* and *TET3* mRNA levels (Fig. 2A). As mentioned above, cultivation with an induction medium without decidualization inducers inhibited metabolic activity in ESCs. We therefore investigated how our selected protocols affected expression of metabolism-associated genes (Fig. 2B). We confirmed that decidualization induction remarkably increased *PPARGC1A* expression, which decreased slightly when 50 IU/ml hCG was added. Additionally, 10 IU/ml hCG decreased *GPX1* expression, while a 50-IU/ml hCG concentration inhibited *TXN* expression in decidualized ESCs. Meanwhile, we did not confirm any significant changes in the expression of *SOD2* (Fig. 2B) as well as implantation process-related genes, such as *MSX1*, *MSX2*, *MMP3*, and *HMX3*, due to either hCG or decidualization induction treatments of ESCs (Supplementary Fig. S1D). These findings confirmed that hCG modulates various aspects of epigenetic regulation in ESCs, including histone modifications and DNA methylation, as well as an impact on metabolic activity.

**Figure 3.**
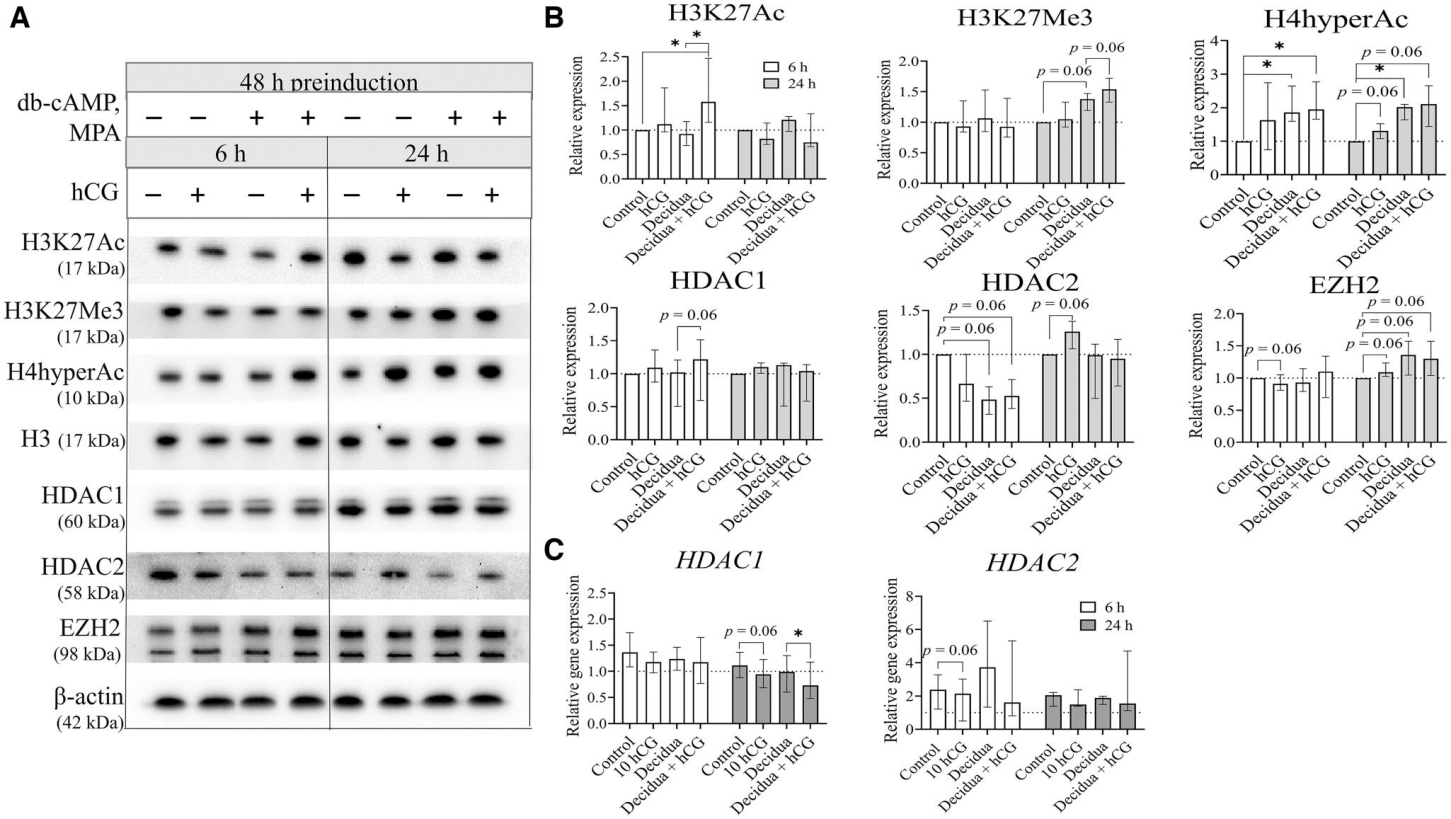
**Changes in histone modifications and their modifiers after decidualization and hCG treatment of ESCs.** (**A**) Representative western blot images of analyzed proteins band intensities. (**B**) Quantification of protein levels from western blot replicates, normalized according to the levels of β-actin (for modifiers), H3, or H4 (for histone modifications). (**C**). RT-qPCR analysis of HDAC1 and HDAC2 expression levels. Results are expressed as fold changes relative to control cells and are presented as the median with interquartile range (n = 4). *P-value <0.05; **P-value <0.01. *P*-values were calculated using the Wilcoxon test. ESCs, endometrial stromal cells.

**Figure 4.**
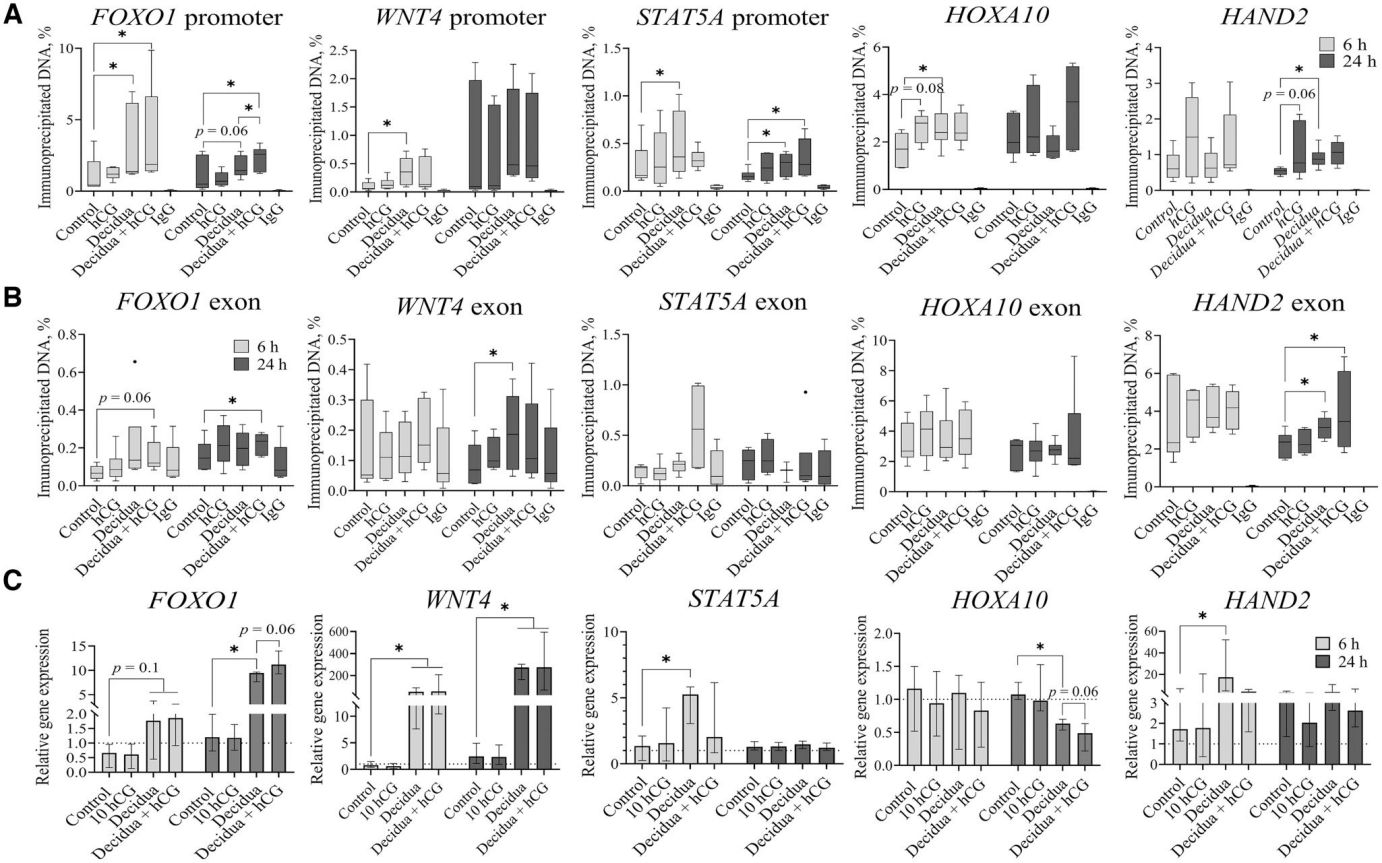
**H3K27Ac modification levels in genes associated with decidualization and implantation regions and expression of these genes after decidualization and hCG treatment of ESCs.** (**A** and **B**) The amount of immunoprecipitated DNA from (A) promoter regions and (B) exon regions. The results are presented as box plots, where the middle line represents the median value, the box represents the interquartile range, and error bars extend to the most extreme data points that are not considered outliers (n = 6). (**C**) RT-qPCR analysis of gene expression. Results are expressed as fold changes relative to control cells and are presented as the median with interquartile range (n = 4). *P-value <0.05; **P-value <0.01. *P*-values were calculated using the Wilcoxon test. ESCs, endometrial stromal cells.

For further analysis, we selected only 10 IU/ml hCG and analyzed its effect on the levels of histone modifications and the proteins catalyzing these modifications in non-decidualized and decidualized ESCs after 6 and 24 h, using immunoblot analysis. Representative images of the bands are presented in Fig. 3A, while quantified results of these immunoblot repeats are shown in Fig. 3B. Our results revealed that 6 h of hCG treatment in decidualized ESCs increased the global levels of active chromatin state markers, including H3K27Ac (Fig. 3B), as well as H3K9Ac and H3KMe3 (Supplementary Fig. S2). However, these increases were not observed after 24 h of treatment. We confirmed that decidualization increased H4hyperAc levels (Fig. 3B), which were further elevated after 24 h of hCG treatment (*P* = 0.06). Additionally, we observed an increase in the global levels of the repressive chromatin mark H3K27Me3 (Fig. 3B) after 24 h of hCG treatment in decidualized ESCs (*P* = 0.06). Next, we analyzed changes in histone modification-catalyzing factors. We found that hCG treatment led to a slight increase in HDAC1 levels after 6 h in decidualized ESCs (*P* = 0.06), while HDAC2 expression was downregulated. However, HDAC2 levels increased after 24 h in non-decidualized cells (*P* = 0.06), while HDAC1 did not significantly differ from the control. Moreover, we demonstrated that 10 IU/ml hCG also downregulated *HDAC1* expression in non-decidualized (*P* = 0.06) and decidualized ESCs after 24 h of treatment (Fig. 3C). On the other hand, we did not confirm any significant changes in *HDAC2* expression, although a decreasing trend was observed after treatment with 10 IU/ml hCG after 6 h, and after 24 h, it returned to the control gene expression level. We also observed slight variations in the levels of EZH2 (Fig. 3B) and EED, while the level of SUZ12 was not affected (Supplementary Fig. S2A). To assess changes in H3K27Ac modification levels at specific gene regions, we performed chromatin immunoprecipitation and extracted DNA fragments associated with this histone modification (Fig. 4). We found that decidualization increased H3K27Ac levels in *FOXO1* promoter, and these levels were further elevated by hCG treatment (Fig. 4A). We demonstrated that *WNT4*, *STAT5A*, *HOXA10*, and *HAND2* promoter regions were enriched with H3K27Ac modification after the decidualization stimulus as well. Moreover, hCG treatment, independent of the decidualization stimulus, led to increased H3K27Ac levels in the promoters of implantation-associated genes, such as *HOXA10* and *HAND2* (*P* = 0.06). Next, we observed that H3K27Ac modification increased in *FOXO1*, *WNT4*, and *HAND2* exon regions after decidualization treatment (Fig. 4B). However, H3K27Ac modification did not significantly differ in endometrium function-associated genes exon regions after hCG treatment. Additionally, we assessed the impact of the observed increase in active chromatin marks on the expression of the analyzed genes (see Fig. 4C). While the changes in the expression of *FOXO1*, *WNT4*, and *STAT5A* correspond with the detected epigenetic alterations, the elevated level of H3K27Ac in the promoter and exon regions of *HOXA10* and *HAND2* does not align with an increase in the expression of these genes. After 6 h, *HAND2* expression increased, while *HOXA10* gene expression decreased after 24 h of treatment. In summary, we demonstrated that hCG treatment initially induced a temporary increase in various active chromatin marks. Subsequently, it increases both active and repressive chromatin marks globally, such as H4hyperAc and H3K27Me3. Moreover, the specific impact of hCG on decidualization (*FOXO1, WNT4, STAT5A*) and implantation processes (*HOXA10*, *HAND2*) occurs through increased H3K27Ac levels in the promoter regions of these genes. While we did not observe a direct impact of these epigenetic modifications on the expression of *HOXA10* and *HAND2* at analyzed time-points, it indicates that these epigenetic marks prepare the cells by priming these genes for a potential increase in later expression.

### hCG impact on ESC-EVs protein and miRNA

**Figure 5.**
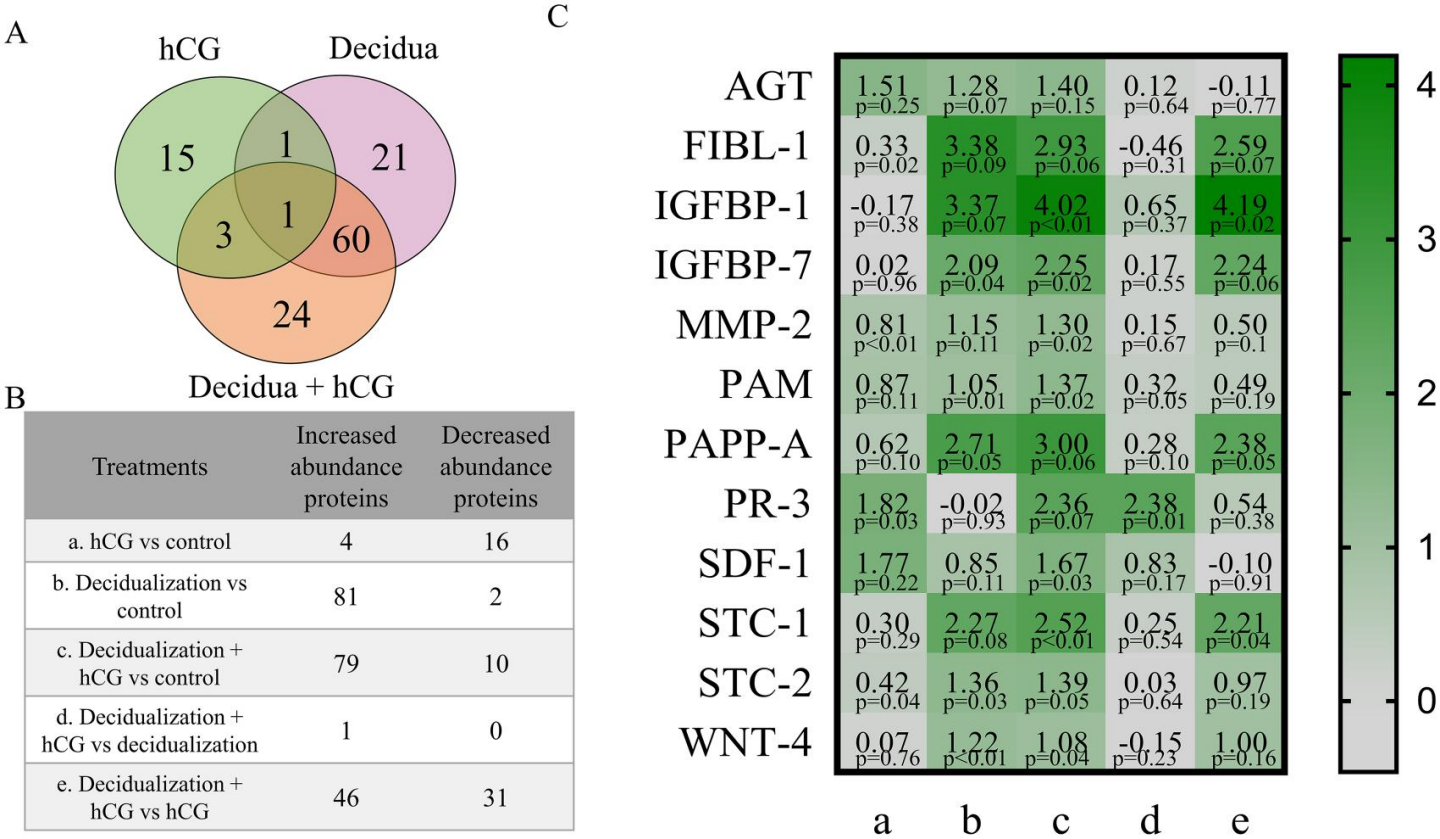
**Protein level changes in ESC-derived EVs following decidualization and hCG treatment assessed by mass spectrometry.** (**A**) Venn diagrams illustrating differentially increased or decreased proteins across treatments. (**B**) A total number of significantly upregulated or downregulated proteins after each treatment, including only those with a *P*-value <0.1 and Log_2 _(fold change) >1.2, was consistently detected in two independent biological replicates. (**C**) Log_2_ (fold change) of pregnancy- and inflammatory response-related proteins following each treatment. Normalized values were used for statistical analysis, and group comparisons were performed using a paired *t*-test. a: hCG vs control; b: Decidualization vs control; c: Decidualization+hCG vs control; d: Decidualization+hCG vs decidualization; e: Decidualization+hCG vs hCG. ESCs, endometrial stromal cells; EVs, extracellular vesicles.

In the next part of the experiments, we analyzed the impact of decidualization and hCG treatment alone and in combination on the protein and miRNA profiles of ESC-secreted EVs. EVs were collected by ultracentrifuging the conditioned medium of treated ESCs. Collected EVs were characterized according to the morphology, size distribution, and protein expression to confirm their identity (Supplementary Fig. S3). Next, we analyzed ESC-EVs’ differentially abundant proteins after decidualization and hCG treatments. Results revealed that decidualization stimulus led to the most changes in protein levels in ESC-EVs (Fig. 5A and B, Supplementary Table S3). hCG treatment led to increased abundance of only a few proteins, while 16 proteins were downregulated in EVs in non-decidualized cells. By analyzing increased levels of proteins in ESC-EVs occurring after decidualization stimulus, we found out that these proteins are involved in embryo implantation, pregnancy, extracellular matrix organization, angiogenesis, and immune response (Table 1, Supplementary Fig. S4). Furthermore, after hCG treatment in non-decidualized ESCs, downregulated proteins in EVs were associated with mitochondrial organization and oxidative phosphorylation (Table 2, Supplementary Fig. S5). After decidualization, increased abundance of proteins associated with pregnancy and embryo implantation are AGT, FIBL-1, IGFBP-1, IGFBP-7, MMP-2, PAM, PAPP-A, STC-1, STC-2, and WNT-4. The changes in the levels of these proteins were compared through all analyzed treatments (Fig. 5C). All of these proteins were significantly upregulated after decidualization or decidualization and hCG treatment compared with the control, while only AGT level was increased after hCG treatment compared with the control. However, none of these proteins were increased after hCG treatment in decidualized ESCs, compared with decidualized cells. PR-3 was the only significantly upregulated protein comparing decidualization and hCG treatment with only decidualization, and we also found that this protein level was increased after hCG and decidualization and hCG treatment compared with the control, while decidualization stimulus alone did not have any effect. Furthermore, we identified that SDF-1 abundance was also increased after hCG and decidualization and hCG combined treatment compared to the control; however, analysis of decidualization compared with the control, or decidualization and hCG compared to only decidualized cells, did not reveal any changes. Comparison of effects caused by combined decidualization and hCG treatment with hCG treatment alone, revealed that decidualization significantly increased the abundance of FIBL-1, IGFBP-1, IGFBP-7, PAPP-A, and STC-1 proteins. Thus, we demonstrated that decidualization stimulus leads to upregulation of embryo implantation and pregnancy-associated proteins in ESC-EVs, while the hCG effect for non-decidualized ESCs is associated with downregulation of proteins involved in oxidative phosphorylation and mitochondrial transport, and specifically led to upregulation of PR-3 and SDF-1.

**Table 1.** The biological processes and functional pathways related to the change in protein abundance of ESC-EVs after decidualization.

Gene ontology (GO) (biological process)			
Number	Description	Count in network	False discovery rate
GO:0030198	Extracellular matrix organization	26 of 278	5.96e−23
GO:0110011	Regulation of basement membrane organization	5 of 11	2.79e−06
GO:0006959	Humoral immune response	9 of 268	0.0005
GO:0038030	Non-canonical Wnt signaling pathway via MAPK cascade	2 of 6	0.0238
GO:0032964	Collagen biosynthetic process	3 of 10	0.0020
GO:0007566	Embryo implantation	4 of 42	0.0038
GO:0007565	Female pregnancy	7 of 170	0.0012
GO:0045765	Regulation of angiogenesis	9 of 288	0.0007
GO:0045087	Innate immune response	11 of 754	0.0194

KEGG analysis			
Number	Description	Count in network	False discovery rate
hsa04512	ECM–receptor interaction	17 of 88	2.80e−20
hsa04610	Complement and coagulation cascades	5 of 82	0.0008
hsa04926	Relaxin signaling pathway	6 of 126	0.0006
hsa04151	PI3K-Akt signaling pathway	15 of 349	1.54e−09

The results were generated using STRING (https://string-db.org/). ESC-EVs, endometrial stromal cells derived extracellular vesicles.

**Table 2.** The biological processes and functional pathways related to the change in protein abundance of ESC-EVs after hCG treatment.

GO (Biological Process)			
Number	Description	Count in network	False discovery rate
GO:0006119	Oxidative phosphorylation	4 of 122	0.0174
GO:0007005	Mitochondrion organization	6 of 445	0.0128

KEGG analysis			
Number	Description	Count in network	False discovery rate
hsa00190	Oxidative phosphorylation	4 of 128	0.0012

The results were generated using STRING (https://string-db.org/). ESC-EVs, endometrial stromal cells derived extracellular vesicles.

**Figure 6.**
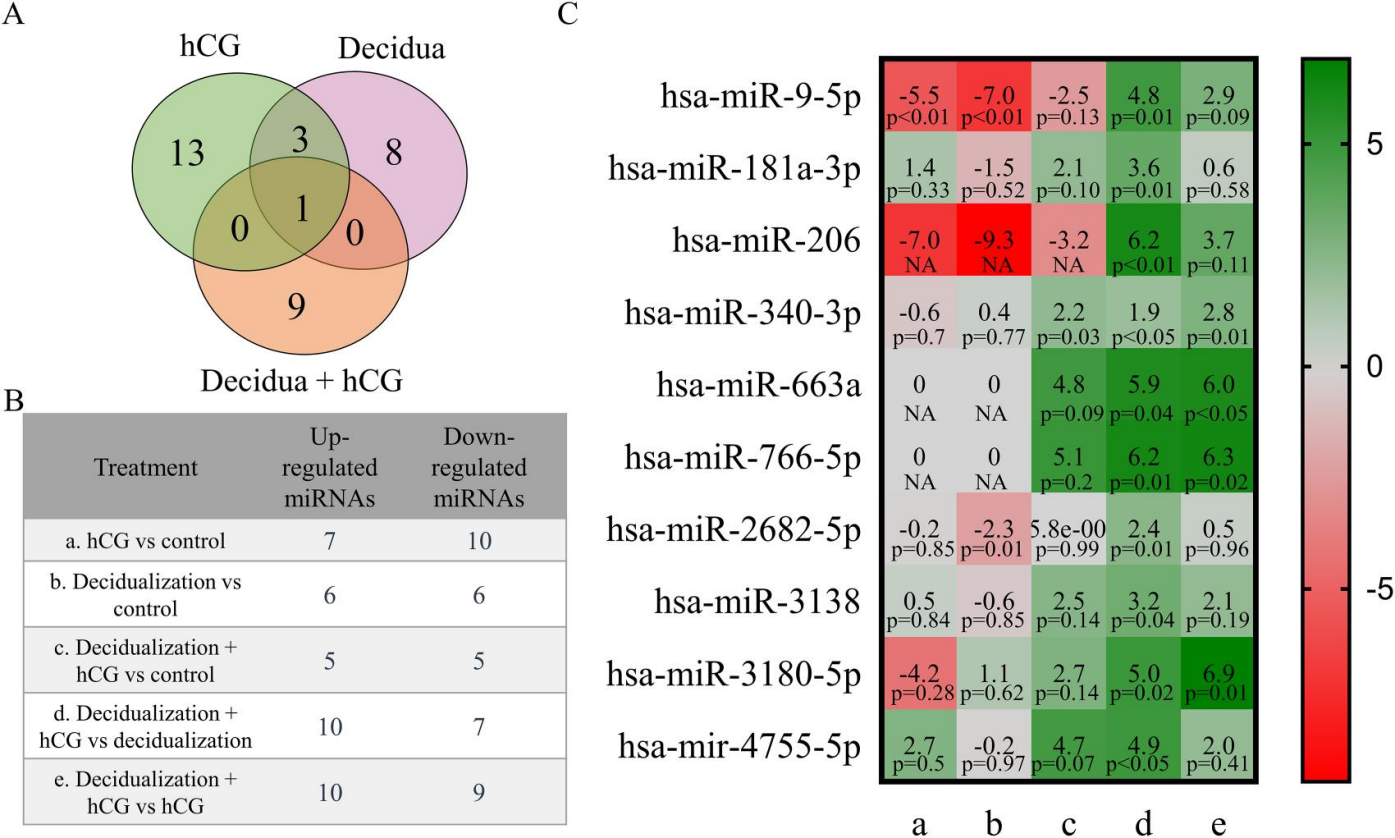
**miRNAs level changes in ESC-derived EVs following decidualization and hCG treatments assessed by small RNA sequencing.** (**A**) Venn diagram illustrating differentially upregulated or downregulated miRNAs across treatments. (**B**) Total number of significantly upregulated or downregulated miRNAs after each treatment, including only those with a *P*-value <0.05 and Log_2 _(fold change) >1.3, which were consistently detected in three independent biological replicates. (**C**) Log_2_ (fold change) of pregnancy-related miRNAs following each treatment. The *P*-values were adjusted using the Benjamini & Hochberg method. a: hCG vs control; b: Decidualization vs control; c: Decidualization+hCG vs control; d: Decidualization+hCG vs decidualization; e: Decidualization+hCG vs hCG. miRNAs, micro-ribonucleic acids; ESCs, endometrial stromal cells; EVs, extracellular vesicles.

Moreover, we assessed the ESC-EVs’ miRNA profiles after decidualization and hCG treatments. miRNA sequencing analysis revealed that most of the significantly differentially expressed miRNAs were unique for each treatment (Fig. 6A and B, Supplementary Table S4). By analyzing after decidualization stimulus differentially expressed miRNAs in ESC-EVs, we determined that these miRNAs are involved in an intracellular signaling cassette in which a small monomeric GTPase relays a signal, actin filament organization, lysosomes, and lysine degradation (Table 3). Furthermore, after hCG treatment in non-decidualized ESCs, differentially expressed miRNAs in EVs were associated with autophagy, regulation of GTPase activity, and histone H4 acetylation (Table 4). Finally, the results of our study revealed that a combination of decidualization and hCG treatments was associated with embryonic organ development, *in utero* embryonic development, and other processes (Tables 5 and 6).

**Table 3.** Association of differentially expressed miRNAs in ESC-EVs following decidualization compared to control with biological and functional processes.

GO (Biological Process)			
Number	Description	Count in network	False discovery rate
GO:0007264	An intracellular signaling cassette in which a small monomeric GTPase relays a signal	229 of 5641	<0.001
GO:0007015	Actin filament organization	156 of 5641	0.002
GO:0007229	Integrin-mediated signaling pathway	53 of 5641	0.025

KEGG analysis			
Number	Description	Count in network	False discovery rate
hsa04142	Lysosome	68 of 2746	0.032
hsa00310	Lysine degradation	35 of 2746	0.049

miRNAs, micro-ribonucleic acids; ESC-EVs, endometrial stromal cells derived extracellular vesicles.

**Table 4.** Association of differentially expressed miRNAs in ESC-EVs following hCG treatment compared to control with biological and functional processes.

GO (Biological Process)			
Number	Description	Count in network	False discovery rate
GO:0006914	Autophagy	153 of 4059	0.022
GO:0043087	Regulation of GTPase activity	154 of 4059	0.043
GO:0043967	Histone H4 acetylation	30 of 4059	0.045

miRNAs, micro-ribonucleic acids; ESC-EVs, endometrial stromal cells derived extracellular vesicles.

**Table 5.** Association of differentially expressed miRNAs in ESC-EVs following decidualization and hCG treatment compared to control with biological and functional processes.

GO (Biological Process)			
Number	Description	Count in network	False discovery rate
GO:0042176	Regulation of protein catabolic process	109 of 3665	0.006
GO:0048732	Gland development	130 of 3665	0.009
GO:0007178	Cell surface receptor protein serine/threonine kinase signaling pathway	99 of 3665	0.009
GO:0010720	Positive regulation of cell development	144 of 3665	0.01
GO:0048568	Embryonic organ development	125 of 3665	0.02
GO:0030111	Regulation of Wnt signaling pathway	98 of 3665	0.04

KEGG analysis			
Number	Description	Count in network	False discovery rate
hsa04072	Phospholipase D signaling pathway	53 of 1816	0.04
hsa01230	Biosynthesis of amino acids	30 of 1816	0.04
hsa04010	MAPK signaling pathway	92 of 1816	0.04

miRNAs, micro-ribonucleic acids; ESC-EVs, endometrial stromal cells derived extracellular vesicles.

**Table 6.** Association of differentially expressed miRNAs in ESC-EVs following decidualization and hCG treatment compared to decidualization with biological and functional processes.

GO (Biological Process)			
Number	Description	Count in network	False discovery rate
GO:0016055	Wnt signaling pathway	264 of 7470	<0.001
GO:0043087	Regulation of GTPase activity	263 of 7470	<0.001
GO:0006914	Autophagy	252 of 7470	0.003
GO:0001701	*In utero* embryonic development	170 of 7470	0.03
GO:0043966	Histone H3 acetylation	38 of 7470	0.04

KEGG analysis			
Number	Description	Count in network	False discovery rate
hsa04919	Thyroid hormone signaling pathway	77 of 3679	0.008
hsa04921	Oxytocin signaling pathway	88 of 3679	0.04
hsa04210	Apoptosis	80 of 3679	0.04
hsa04010	MAPK signaling pathway	161 of 3679	0.04
hsa04910	Insulin signaling pathway	78 of 3679	0.04

miRNAs, micro-ribonucleic acids; ESC-EVs, endometrial stromal cells derived extracellular vesicles.

For further evaluation, we selected after hCG treatment in decidualized ESC-EVs (decidualization+hCG vs decidualization) significantly upregulated miRNAs, and changes in these miRNA levels after all analyzed treatments were compared (Fig. 6C). The levels of hsa-miR-9-5p and hsa-miR-206 decreased after decidualization and hCG alone or combination treatment compared to the control. However, levels of hsa-miR-9-5p and hsa-miR-206 were increased when decidualized ESCs were treated with hCG compared to only decidualized cells. hsa-miR-181a-3p expression was increased after hCG and decidualization with hCG compared to the control, as well as decidualization with hCG compared to only decidualized cells, while decidualization stimulus by itself led to downregulation of this miRNA. hsa-miR-340-3p, hsa-miR-663s, hsa-miR-766-5p, hsa-miR-3138, and hsa-miR-3180-5p changes were not detected after decidualization or hCG treatment separately; however, the combination of both these treatments led to a more than 2-fold increase of these miRNAs compared with the control or with decidualization. Additionally, hsa-miR-340-36p and hsa-miR-3180-5p showed a significant increase following decidualization, which was confirmed by comparing the effects of decidualization and hCG with those of hCG treatment alone. The decreased amount of hsa-miR-2682-5p was detected after decidualization treatment; however, when decidualized ESCs were treated with hCG, this miRNA level increased more than two times compared with only decidualized cells. The hsa-miR-4755-5p level increased after hCG treatment in both non-decidualized and decidualized ESCs, while decidualization had a negative effect on this miRNA level. So decidualization and hCG, and these treatments in combination, lead to various miRNA level changes in ESC-EVs; however, hCG treatment of decidualized ESCs specifically increased levels of such miRNAs as hsa-miR-340-3p, hsa-miR-663a, hsa-miR-766-5p, hsa-miR-3138, hsa-miR-3180-5p, and hsa-miR-4755-5p.

## Discussion

In the study, we examined the effects of decidualization and one of the first embryo-secreted factors, hCG, which signals the endometrium about the presence of an embryo in the preimplantation period (d’Hauterive *et al.*, 2022), on ESC changes. We found that the hCG at concentrations of 10 and 50 IU/ml did not negatively affect the metabolic activity and viability of ESCs. However, higher concentrations of hCG resulted in inhibition of cellular metabolic activity. A previous study focusing on ovarian cells indicated that hCG concentrations up to 10 IU/ml also did not have a negative effect on cell viability. Moreover, treatment with 100 IU/ml hCG increased cell viability. The authors of that study suggested that hCG treatment in combination with progesterone increases ovarian cell survival. As the progesterone level decreases during the menstrual cycle, this can lead to apoptosis in ovarian cells (Hirata *et al.*, 2015). Therefore, the effect of hCG on cell viability is influenced by various factors such as the type of cells, the concentration of hCG, and the hormonal background at that time.

Next, we investigated the dose-dependent effects of hCG treatment on the expression of markers associated with the decidualization process. Our findings revealed that a concentration of 10 IU/ml hCG resulted in the most significant increase in prolactin expression. In contrast, a higher concentration led to a lower, yet still significant, increase. Importantly, this increase was observed only in previously decidualized ESCs, as hCG did not influence prolactin expression in non-decidualized cells. These results are consistent with physiological processes *in vivo*, as endometrial stroma begins to decidualize after ovulation, so by the time the embryo reaches the uterus and starts to secrete hCG, the ESCs have already started their decidualization process (Ochoa-Bernal & Fazleabas, 2020). Thus, our results confirm that hCG is not a primary inducer of decidualization but rather an enhancer of this process. Previously, the positive impact of hCG treatment on decidualization was demonstrated using a mouse model. When human endometrial tissue fragments were transplanted, and hCG was injected, an increase in the area undergoing decidualization, as well as an increase in expression of decidualization markers, was observed (Koch *et al.*, 2018). Another study showed that treatment with 10 IU/ml of hCG successfully increased intracellular cAMP levels and induced Erk1/2 phosphorylation (Tapia-Pizarro *et al.*, 2017). Additionally, higher concentrations of hCG, such as 500 IU/ml, inhibited estrogen and progesterone-induced IGFBP-1 synthesis and secretion (Fleming *et al.*, 2008). Based on our findings, the supplementary positive impact of hCG treatment on the decidualization process depends on the concentration used, with 10 IU/ml demonstrated to be the most beneficial effect among the concentrations tested in this study.

Through our analysis of the impact of hCG on histone modifications in ESCs, we found that short-term treatment with hCG (6 h) significantly increased global levels of active chromatin marks, specifically histone modifications such as H3K27Ac, H3K4Me3, and H3K9Ac. We observed a temporary decrease in gene expression and protein levels of the histone deacetylase HDAC2 after 6 h of hCG treatment, while HDAC1 exhibited less markable changes, including decreased gene expression with a slight increase or no changes in protein level. Discrepancies between gene expression and protein levels can arise from various factors, such as post-translational modifications, protein stability, and protein turnover rates (Andrieux *et al.*, 2020). Although it is known that HDAC1 and HDAC2 have similar functions in epigenetic regulation and their roles vary depending on cell type and developmental processes (Ma and Schultz, 2016), our study highlights the relationship between decidualization, hCG treatment, and changes in HDAC2 expression in ESCs. Additionally, we noted an increase in the level of histone H3K27Ac modification, which may be related to the decrease in HDAC2 protein levels. A recent study indicated that even a shorter exposure to hCG (1–2 h) resulted in elevated levels of phosphorylated HDAC2, leading to global deacetylation of the genome. However, this deacetylation was subsequently replaced by the establishment of a specific histone acetylation profile after about 4 h. Notably, the study demonstrated a particular increase in H3K27Ac levels in genes related to histone H3 acetylation, positive regulation of transcription, mRNA synthesis, and translation (Jin *et al.*, 2023). Our research further revealed that hCG treatment raised the H3K27Ac levels in regions associated with genes involved in decidualization (such as *FOXO1*) and implantation (including *HOXA10* and *HAND2*). For FOXO1, the increase from the decidualization effect was further enhanced by hCG treatment. This finding confirms our earlier observations regarding the positive impact of hCG on decidualization. Importantly, hCG also exerted an independent effect on the histone acetylation levels of implantation-related genes. HOXA10 is known to be a regulator of endometrial receptivity (Sahar *et al.*, 2019). Previous research indicated that the injection of hCG into the uterus leads to an increase in *HOXA10* gene expression (Zhu *et al.*, 2020). Similarly, HAND2 is a crucial implantation marker, as it regulates the balance of estrogen and progesterone receptors necessary for successful embryo implantation (Sucurovic *et al.*, 2020). Therefore, the changes in global histone acetylation induced by hCG modulate cellular responses required for tissue functionality, while specific effects are mediated through temporally decrease of HDAC2 and increases in H3K27Ac levels in implantation and decidualization-related gene regions.

EVs play a crucial and dynamic role in transmitting information between different biological compartments. Their significance in mediating communication between the embryo and maternal tissues has been demonstrated through their capacity to regulate receptivity, implantation, and trophoblast invasion (Hart *et al.*, 2022; Sui *et al.*, 2023). In our study, we evaluated how decidualization and the impact of hCG affected the proteomic profiles of ESC-EVs. We found that the stimulus of decidualization led to the most significant changes, overshadowing the effects of hCG. Decidualization treatment increased ESC-EV proteins associated with embryo implantation, pregnancy, and various biological processes, such as extracellular matrix remodeling, angiogenesis, and immune response regulation. Previous studies have also confirmed that the cargo of endometrial EVs regulates tissue reorganization, angiogenesis, antioxidant, and immunosuppressive functions, as well as the migration and invasiveness of trophoblast cells (Fatmous *et al.*, 2022; Sui *et al.*, 2023). Our study identified several proteins with significantly altered abundance in ESC-EVs following decidualization, including AGT, FIBL-1, IGFBP-1/7, MMP-2, STC-1/2, PAM, PAPP-A, and WNT-4. AGT is the precursor of angiotensin and plays a role in regulating blood pressure, salt balance, and fluid homeostasis (Lumbers & Pringle, 2014). FIBL-1 and MMP2 are proteins that regulate the extracellular matrix and are highly expressed in placental tissue (Nikolov and Popovski, 2021; Orvik *et al.*, 2021). IGFBP-1 is a well-known marker of the decidualization process, primarily functioning to bind insulin-like growth factors, thereby regulating their biological activity and prolonging their half-life (Hivert *et al.*, 2024). Additionally, a decrease in IGFBP-7 levels has been linked to recurrent spontaneous pregnancy loss and trophoblast invasiveness dysfunction (Wu *et al.*, 2022). The level of STC-1, a hormone with pleiotropic functions, increases during early pregnancy and is also associated with the regulation of IGF bioavailability (Bishop *et al.*, 2021; Conover and Oxvig, 2023). During hCG treatment, the impact on ESC-EV protein abundance changes was less pronounced; however, we observed an increase in the levels of protease PR-3 and the chemokine SDF-1. PR-3 is a serine protease involved in granulocyte differentiation and is predominantly expressed in neutrophils (Akgun *et al.*, 2020). SDF-1 is a significant factor in establishing a pro-inflammatory environment, as it activates various leukocytes (Janssens *et al.*, 2018). SDF-1 is also recognized as an implantation marker since its expression increases following hCG treatment (Freis *et al.*, 2019). In summary, after the decidualization stimulus, we observed an increase in ESC-EVs of proteins critical for embryo implantation and subsequent development. These changes are associated with the regulation of angiogenesis, extracellular matrix reorganization, and IGF signaling in targeted cells. Alternatively, the effects of hCG treatment resulted in elevated levels of factors involved in immune system regulation.

We assessed the impact of decidualization and hCG treatment on the miRNA profile of ESC-derived EVs. Our analysis revealed that most differentially expressed miRNAs were unique to each treatment, with only a few significantly altered miRNAs being common between the two analyzed treatments. Our study confirmed that the combination of decidualization and hCG treatment led to increased levels of several miRNAs, including hsa-miR-340-3p, hsa-miR-663a, hsa-miR-766-5p, hsa-miR-3138, hsa-miR-3180-5p, and hsa-miR-4755-5p. A recent study has shown that EVs containing miR-340-3p enhance endometrial recovery after injury in rats by inhibiting ferroptosis (Xiao *et al.*, 2024). Moreover, a decreased level of miR-766-5p has been associated with recurrent pregnancy loss (Xu *et al.*, 2022). Additionally, miR-3138 has been found to contribute to insulin resistance in human umbilical vein endothelial cells by indirectly inhibiting glucose transporter molecules (Chen *et al.*, 2021). Furthermore, hsa-miR-4755-5p has been detected in successfully developing blastocysts and is considered a potential biomarker for identifying high-quality blastocysts (Pasquariello *et al.*, 2024). No studies have identified effects on the reproductive system of the other differentially expressed EV miRNAs reported here. However, the significance of these miRNAs in other body systems has been confirmed. For instance, in skin cells responsible for hair growth, miR-3180-5p has been shown to inhibit the Bax protein, thus promoting apoptosis (Wei *et al.*, 2023). Additionally, miR-663a inhibits TGF, reducing cell migration and the epithelial-to-mesenchymal transition (Qu *et al.*, 2022). Thus, the increased levels of these miRNAs in EVs after decidualization and hCG treatment in ESCs may have varying effects depending on the environmental conditions. Further research could complement and expand our findings to accurately determine the influence of these miRNAs on the implantation process within the endometrial environment.

In conclusion, our results indicate that hCG significantly enhanced the decidualization process in ESCs, leading to an elevation in decidualization marker expression. Furthermore, exposure to hCG increases the levels of H3K27Ac modifications in gene regions associated with decidualization (such as *FOXO1*) and implantation (including *HOXA10* and *HAND2*). After decidualization, the abundance of proteins in ESC-derived EVs related to embryo implantation and pregnancy, such as FIBL-1, IGFBP-1/7, MMP-2, STC-1/2, and PAPP-A, shows an increase. Also, hCG treatment elevates proteins involved in immune system regulation, including PR-3 and SDF-1. Moreover, hCG exposure leads to enhanced levels of specific miRNAs (hsa-miR-340-3p, hsa-miR-663a, hsa-miR-766-5p, hsa-miR-3138, and hsa-miR-3180-5p) in decidualized ESC-EVs. These findings suggest that hCG modulates the molecular environment of decidualized ESCs, potentially influencing implantation and immune regulation through epigenetic modifications, and secreted and miRNA levels.

## Supplementary Material

hoaf051_Supplementary_Data

## Data Availability

Data available on request.
