# Supplementary material for: Evaluating endometrial response to human chorionic gonadotropin: alterations in epigenetic regulation and extracellular vesicle cargo of endometrial stromal cells
Source: Hum Reprod Open. 2025 Aug 14;2025(3):hoaf051. doi: 10.1093/hropen/hoaf051 (PMC12408481; doi:10.1093/hropen/hoaf051)
Supplement: hoaf051_Supplementary_Data [file hoaf051_supplementary_data.zip › Supplementary_Tables_S1-S2_EO.docx]

| **Supplementary Table S1.** Primer sequences used for RT-qPCR | |
| --- | --- |
| **Gene** | **Primer sequence (5′–3′)** |
| *GAPDH* | F’ AGTCCCTGCCACACTCAG |
|  | R’ TACTTTATTGATGGTACATGACAAGG |
| *RPL13A* | F’ GTTGATGCCTTCACAGCGTA |
|  | R’ AGATGGCGGAGGTGCAG |
| *HDAC1* | F’ CAAGCTCCACATCAGTCCTTCC |
|  | R’ TGCGGCAGCATTCTAAGGTT |
| *HDAC2* | F’ AGTCAACGAGGCGGCAAAA |
|  | R’ TGCGGATTCTATGAGGCTTCA |
| *HMX3* | F’ GCCCATCCTCTACCACGAGA |
|  | R’ GTGCGAGTAGTAGACGGGGT |
| *MSX1* | F’ CCACTCGGTGTCAAAGTGGA |
|  | R’ GAAGGGGACACTTTGGGCTT |
| *MSX2* | F’ CCGCCAAGACATATGAGCCC |
|  | R’ GGAGAACTCTGCACGCTCTG |
| *PPARGC1A* | F’ GCAATTGAAGAGCGCCGTGTGA |
|  | R’ CTGTCTCCATCATCCCGCAGAT |
| *BAX* | F’ TGCCTCAGGATGCGTCCACCAA |
|  | R’ CCCCAGTTGAAGTTGCCGTCAG |
| *BAK1* | F’ TCATCGGGGACGACATCAAC |
|  | R’ CAAACAGGCTGGTGGCAATC |
| *BCL2* | F’ CGGAGGCTGGGATGCCTTTG |
|  | R’ TTTGGGGCAGGCATGTTGAC |
| *GPX1* | F’ CAGTCGGTGTATGCCTTCTCG |
|  | R’ GAGGGACGCCACATTCTCG |
| *SUZ12* | F’ AGGCTGACCACGAGCTTTTC |
|  | R’ GGTGCTATGAGATTCCGAGTTC |
| *EED* | F’ GTGACGAGAACAGCAATCCAG |
|  | R’ TATCAGGGCGTTCAGTGTTTG |
| *PRL* | F’ TGACCCTTCGAGACCTGTTTG |
|  | R’ CTTGCTCCTTGTCTTCGGG |
| *IGFBP1* | F’ TTTTACCTGCCAAACTGCAACA |
|  | R’ CCCATTCCAAGGGTAGACGC |
| *IL1B* | F’ AGATGATAAGCCCACTCTACAG |
|  | R’ ACATTCAGCACAGGACTCTC |
| *IL6* | F’ ACAGCCACTCACCTCTTCAG |
|  | R’ CCATCTTTTTCAGCCATCTTT |
| *IL11* | F’ ATGAACTGTGTTTGCCGCCT |
|  | R’ GGGAATCCAGGTTGTGGTCC |
| *TET1* | F’ TTCGTCACTGCCAACCTTAG |
|  | R’ ATGCCTCTTTCACTGGGTG |
| *TET2* | F’ CCCTTCTCCGATGCTTTCTG |
|  | R’ TGGGTTATGCTTGAGGTGTTC |
| *TET3* | F’ TCCAGCAACTCCTAGAACTGAG |
|  | R’ AGGCCGCTTGAATACTGACTG |

| **Supplementary Table S2.** Primer sequences used for ChIP-qPCR | |
| --- | --- |
| **Gene region** | **Primer sequence (5′–3′)** |
| *WNT4* promoter | F’ TCCTCCCAATCACAGCGTCT |
|  | R’ GTGGGAATCCGAAACCTCGC |
| *WNT4* exon | F’ AACTGCTCCACACTCGACTC |
|  | R’ TGCTCACGAGCGTCTCATTT |
| *HAND2* promoter | F’ TGAGGAGGTAGCCAATCCTG |
|  | R’ AGGGCCGCTCGGGTTAATA |
| *HAND2* exon | F’ CAGCTACATCGCCTACCTCA |
|  | R’ TCTCCTCTTTCACGTCGGTC |
| *FOXO1* promoter | F’ GCTCTGCTGCTCCGTAGTAA |
|  | R’ TCTCTCGCCTTCTCAGTGTT |
| *FOXO1* exon | F’ TGGAGTACATTTCGCCCTCG |
|  | R’ AGTAGAGGCCATCTTTGCGG |
| *HOXA10* promoter | F’ ATGTGGTCGTAAACCCGTCC |
|  | R’ CAGCCCGCTGCTATTGAGAT |
| *HOXA10* exon | F’ AAGAGTGGTCGGAAGAAGCG |
|  | R’ GACGCTGCGGCTAATCTCTA |
| *STAT5A* promoter | F’ GGGGCGCTGGCTAGTTTAT |
|  | R’ AAACTCAACCCTGACGGAGG |
| *STAT5A* exon | F’ CACCCGCAAGTAATTGTGCC |
|  | R’ TGGATGCAAGGACAAAGCGG |
| *GAPDH* promoter | F’ TACTAGCGGTTTTACGGGCG |
|  | R’ GGCTGCGGGCTCAATTTATAG |
