# Supplementary material for: Evaluating endometrial response to human chorionic gonadotropin: alterations in epigenetic regulation and extracellular vesicle cargo of endometrial stromal cells
Source: Hum Reprod Open. 2025 Aug 14;2025(3):hoaf051. doi: 10.1093/hropen/hoaf051 (PMC12408481; doi:10.1093/hropen/hoaf051)
Supplement: hoaf051_Supplementary_Data [file hoaf051_supplementary_data.zip › Supplementary-Figs-post_adjudication_clean_EO.docx]

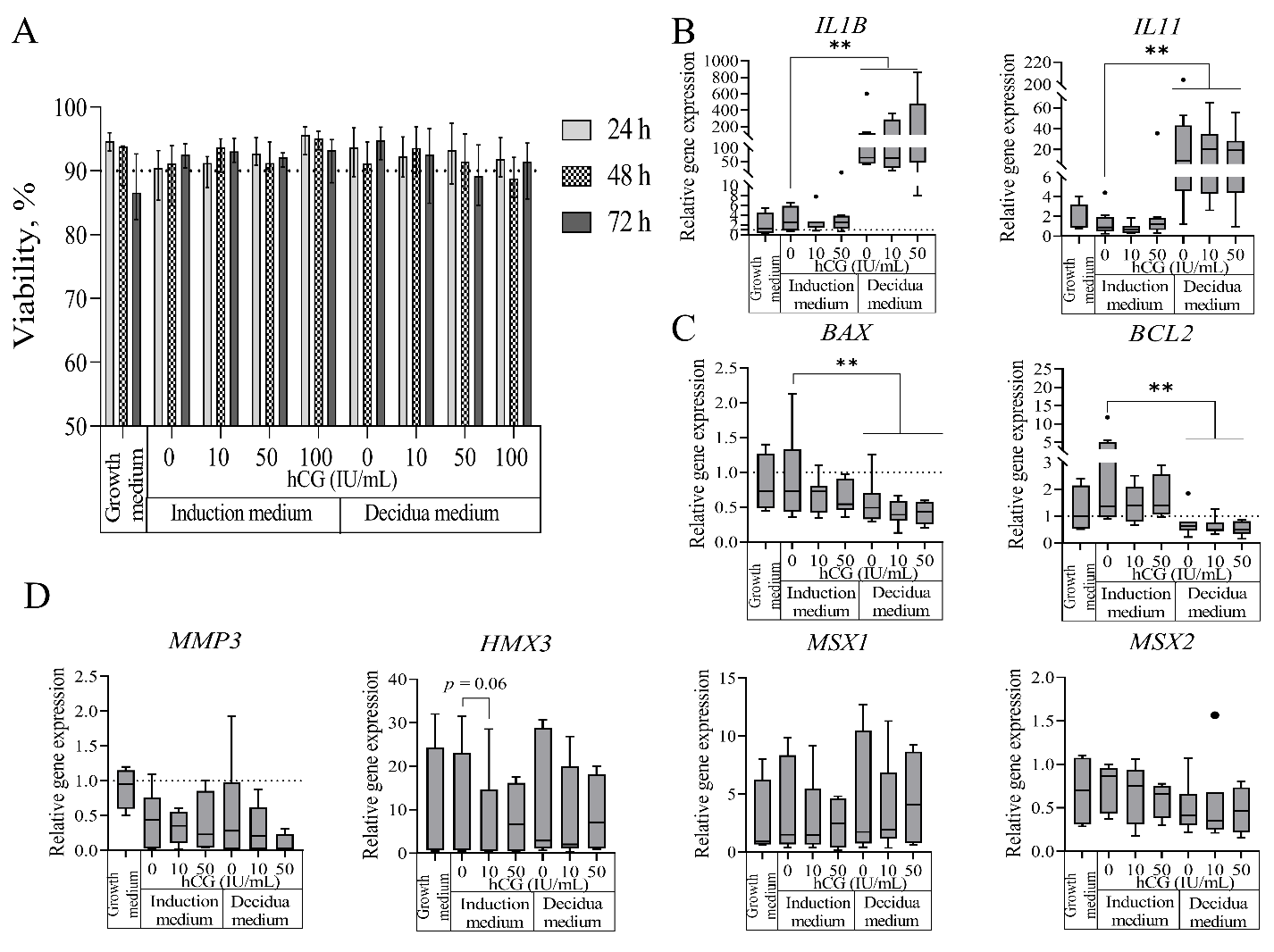


**Supplementary Fig S1. Assessment of hCG impact on ESCs properties.** A. Viability assessment by trypan blue staining (n = 4). B. Interleukins coding gene expression changes. C. Apoptosis-related genes. D. Implantation process associated genes Gene expression changes after decidualization and hCG treatments confirmed by RT-qPCR. Results were calculated using the ΔΔC_T_ method (n = 8). The geometric mean of *GAPDH* and *RPL13A* expression levels was used for mRNA level normalization. Results are presented as box plots, where the middle line represents the median value. p-values were calculated using the Wilcoxon test. ESCs, endometrial stromal cells


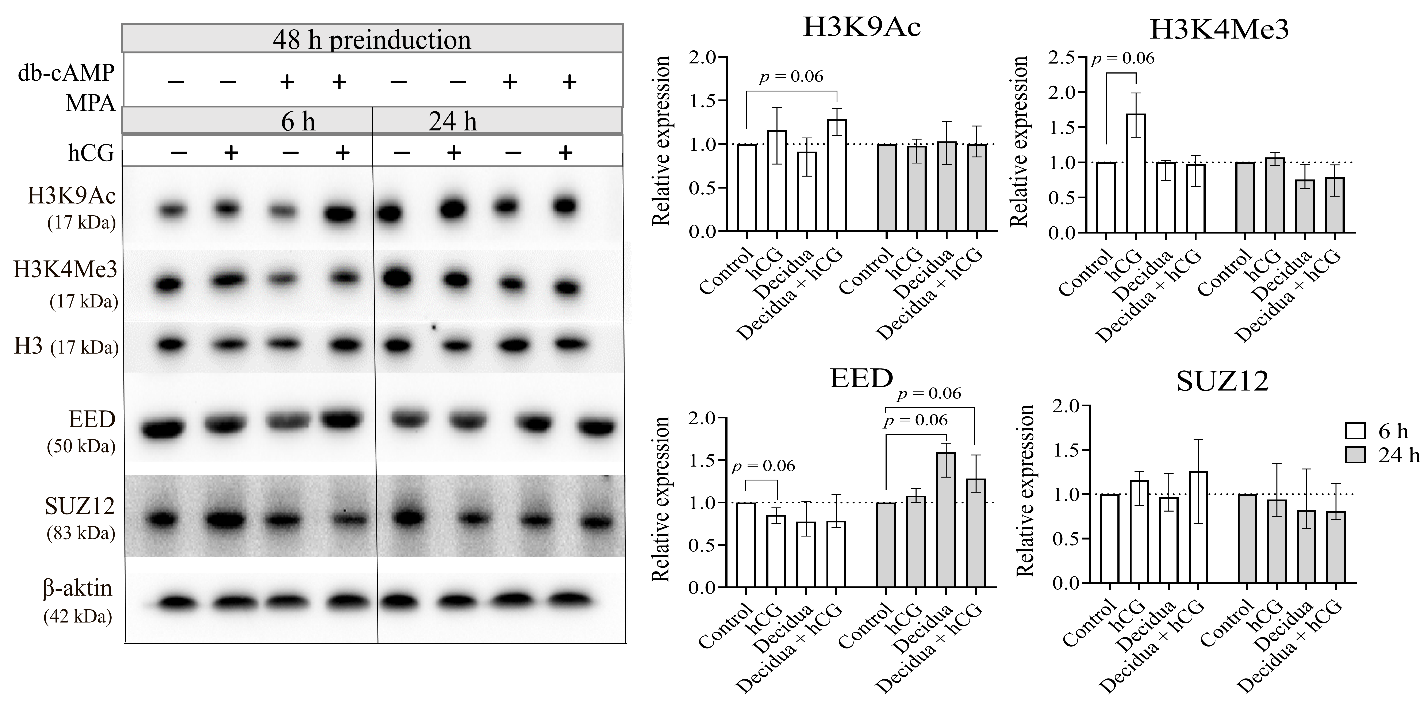


**Supplementary Fig S2. Changes in epigenetic modifications after decidualization and hCG treatment of endometrial stromal cells (ESCs).** Representative and quantification of protein levels from Western Blot replicates, normalized according to the levels of β-actin (for modifiers), H3 (for histone modifications). Results are expressed as fold changes relative to control cells and are presented as the median with interquartile range (n = 4).

**
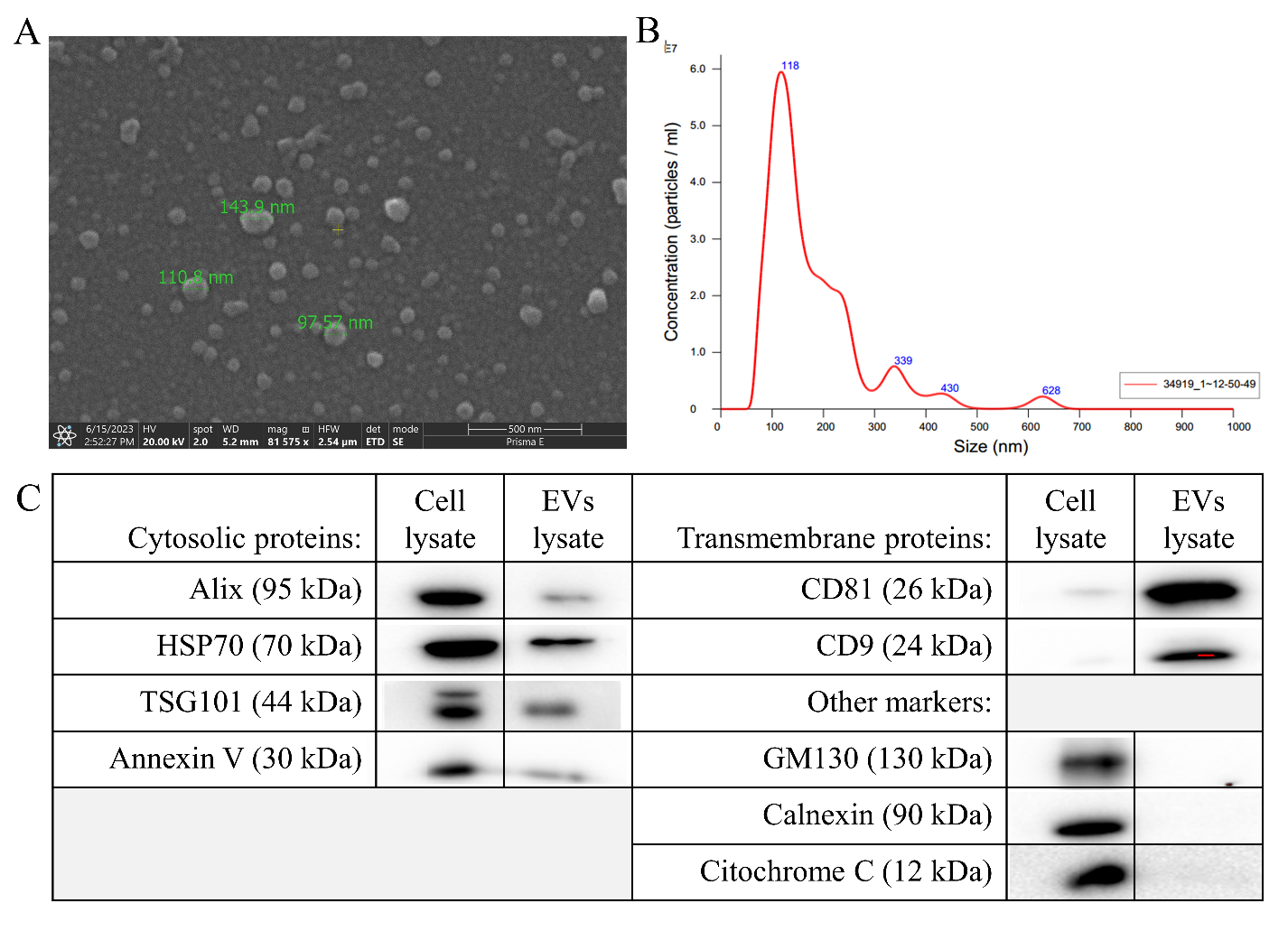
Supplementary Fig S3. Characterization of endometrial stromal cells derived extracellular vesicles (ESC-EVs) collected by ultracentrifugation.** A. Visualization and confirmation of morphology by scanning electron microscope (SEM). B. EVs' size distribution and concentration determination by NanoSizer. C. Protein specific for cell lysates or EVs detection by Western Blot.

**
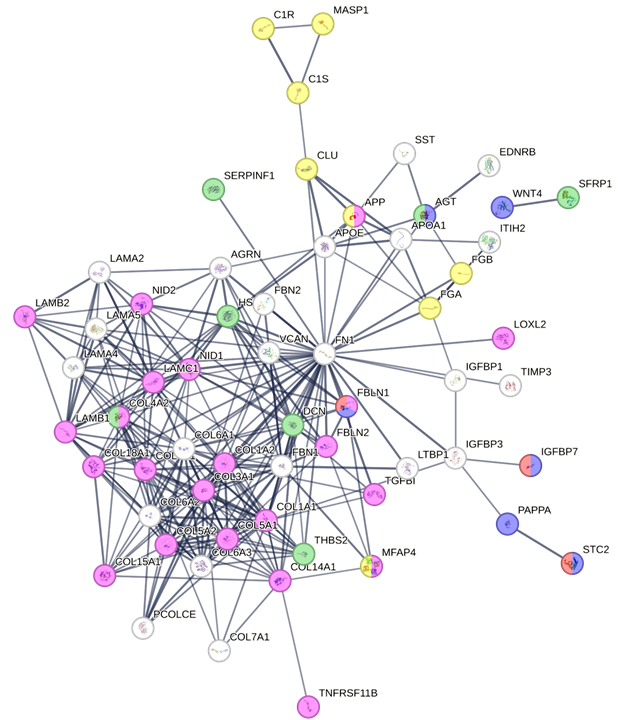
**

**Supplementary Fig S4. After decidualization treatment, differentially abundant proteins of the endometrial stromal cells derived extracellular vesicles (ESC-EVs) interaction network.** The protein interaction network was generated using STRING (<https://string-db.org/>; 2024-06-02). Colours represent proteins associated with the same biological process. Red, embryo implantation (GO:0007566); blue, pregnancy (GO:0007565); purple, extracellular matrix organization (GO:0030198); green, angiogenesis regulation (GO:0045756); yellow, humoral immune response (GO:0006959); grey, other biological processes.


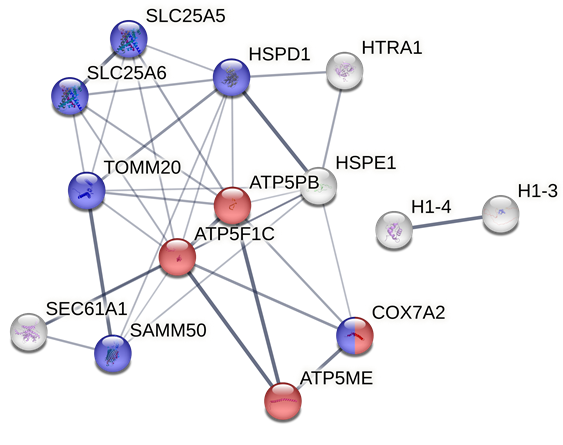
**Supplementary Fig S5. After hCG treatment, differentially abundant proteins of the endometrial stromal cells derived extracellular vesicles (ESC-EVs) interaction network.** The protein interaction network was generated using STRING (<https://string-db.org/>; 2024-06-02). Colours represent proteins associated with the same biological process. Red, oxidative phosphorylation (GO:006119); blue, mitochondrion organization (GO:0007005); grey, other biological processes.
